# VM-YOLO: YOLO with VMamba for Strawberry Flowers Detection

**DOI:** 10.3390/plants14030468

**Published:** 2025-02-05

**Authors:** Yujin Wang, Xueying Lin, Zhaowei Xiang, Wen-Hao Su

**Affiliations:** 1 School of Mechanical Engineering, Chongqing University of Technology, Banan, Chongqing 400054, China; wangyujin@cqut.edu.cn (Y.W.); lxueying0524@163.com (X.L.); xiangzhaowei@cqut.edu.cn (Z.X.); 2 College of Engineering, China Agricultural University, Haidian, Beijing 100083, China

**Keywords:** object detection, state space model, strawberry flower, YOLOv8

## Abstract

Computer vision technology is widely used in smart agriculture, primarily because of its non-invasive nature, which avoids causing damage to delicate crops. Nevertheless, the deployment of computer vision algorithms on agricultural machinery with limited computing resources represents a significant challenge. Algorithm optimization with the aim of achieving an equilibrium between accuracy and computational power represents a pivotal research topic and is the core focus of our work. In this paper, we put forward a lightweight hybrid network, named VM-YOLO, for the purpose of detecting strawberry flowers. Firstly, a multi-branch architecture-based fast convolutional sampling module, designated as Light C2f, is proposed to replace the C2f module in the backbone of YOLOv8, in order to enhance the network’s capacity to perceive multi-scale features. Secondly, a state space model-based lightweight neck with a global sensitivity field, designated as VMambaNeck, is proposed to replace the original neck of YOLOv8. After the training and testing of the improved algorithm on a self-constructed strawberry flower dataset, a series of experiments is conducted to evaluate the performance of the model, including ablation experiments, multi-dataset comparative experiments, and comparative experiments against state-of-the-art algorithms. The results show that the VM-YOLO network exhibits superior performance in object detection tasks across diverse datasets compared to the baseline. Furthermore, the results also demonstrate that VM-YOLO has better performances in the mAP, inference speed, and the number of parameters compared to the YOLOv6, Faster R-CNN, FCOS, and RetinaNet.

## 1. Introduction

The advent of computer vision has led to its extensive application in the agricultural domain, particularly for the purpose of flower detection [1,2,3]. The non-invasive nature of computer vision, which allows for the avoidance of any potential damage to delicate flowers, makes it a crucial technology in the field of flower identification. The rapid and accurate detection of flowers is of paramount importance for tasks including flower thinning, pollination, and yield estimation. Consequently, many studies have focused on computer-vision-based flower detection methods, which can generally be divided into the following two categories: traditional digital image processing [4,5] and machine learning methods [6,7,8].

On the one hand, the majority of traditional digital image processing-based flower detection methods employ image filtering, feature fusion, edge detection, and other algorithms to extract feature information from flower images. For example, Thorp et al. [9] transformed camera-captured images of Lesquerella canopies into hue, saturation, and intensity (HSI) color spaces and then employed a Monte Carlo approach to address uncertainty in the HSI parameters used for image segmentation. Through this digital imaging method, they estimated flower numbers from image-based flower cover percentages; however, they were unable to detect individual flowers because of the complexity of the flowering patterns within the image scene. In a study by Hočevar et al. [10], the HSI thresholding method was also employed to estimate flowering in apple orchards. In order to detect tangerine flowers under natural lighting conditions and then estimate the yield of tangerines in orchards, an algorithm involving white color detection and white color pixel counting was developed by Dorj et al., in which the Gaussian filtering method was employed to mitigate the influences of image noise and illumination [11]. In order to evaluate the flowering statuses of maize and soybean from a series of daily images captured under the same lighting conditions, McCarthy et al. [12] employed an RGB color segmentation method to detect flowers. The flower was identified on the basis of the ratio of the red channel to the green channel. A ratio greater than 0.92 indicated that the pixel was more inclined toward yellow and may represent a flower. Accordingly, the flowering status could be determined by calculating the ratio of yellow pixels to total pixels within the image. Traditional digital image processing methods necessitate a high degree of uniformity in the backgrounds of flower images, resulting in a weak adaptation to lighting variations [13]. Furthermore, the complex conditions that prevail in natural environments, such as occlusion and overlap of flowers, present additional challenges in image processing.

On the other hand, machine learning methods have been extensively employed for flowers detection in recent years because of their strong generalization performance [14,15]. Toğaçar et al. [16] proposed a hybrid method for flower species classification using features extracted from the intersection of feature selection methods in convolutional neural network (CNN) models and achieved an overall accuracy rate of 98.91% for five flower classes. In a study by Cıbuk et al. [17], the features of flowers in images were extracted using the AlexNet and VGG16 models. Subsequently, the more efficient features selected by the minimum redundancy maximum relevance (mRMR) method were used in flower species classification through a support vector machine (SVM) classifier with radial bases function (RBF) kernel. The experimental results demonstrate that this approach can achieve a high level of accuracy despite its apparent simplicity. Tian et al. [18] employed an SSD (single-shot multibox detector) neural network to detect and identify flowers in a video stream, achieving high accuracy and fast detection speed. To accurately detect apple flowers, Wu et al. [19] proposed a real-time apple flower detection method based on a channel-pruned YOLOv4 deep learning algorithm. The experimental results for the apple flower images dataset, for which the images were captured manually in natural environments, demonstrates that this method can effectively reduce the number of parameters, model size, and inference time while maintaining a high mAP of 97.31%, which is only 0.24% lower than the model’s value prior to pruning. In research by Tao et al. [20], an improved CNN model that incorporates an attention mechanism and multi-scale feature fusion was proposed to detect peach flower density to provide visual support for intelligent mechanical flower thinning operations. In order to identify individual king flowers within flower clusters for precision pollination, Mu et al. [21] developed a Mask R-CNN-based detection model followed by a king flower segmentation algorithm to identify and locate king flowers during anthesis, achieving a detection accuracy that varied from 98.7% to 65.6%, with respect to the flower stage, from 20% to 80% blooming. Xu et al. [22] adopted the YOLOv5s model with the Squeeze-and-Excitation (SE) attention mechanism to detect cucumber flowers within greenhouse environments, obtaining a high mAP of 0.847 when classifying cucumber flowers into the following three stages: bud, bloom, and faded flower. Dias et al. [23] proposed a flower detection model based on an end-to-end residual convolutional neural network, which is robust to variations in clutter and illumination due to its integration of both color and morphological information.

Recently, flower detection studies have increasingly concentrated on the accuracy of object detection, leading to considerable achievements in this field. However, the aforementioned methods are usually time-consuming and computationally expensive, which constrains their applicability for mobile agricultural equipment with limited computational resources [24]. The need for more lightweight algorithms remains paramount, despite the efforts of previous studies to reduce the complexity and computational demands of various detection tasks [25,26,27,28,29,30]. Furthermore, there has been a paucity of research dedicated to the detection of small, dense, and occluded objects. In order to meet the requirements of accuracy and lightweight in the task of strawberry flower detection, this paper proposes an object detection algorithm based on the incorporation of YOLOv8 and VMamba (Visual State Space Model) [31]. The main contributions of our work can be outlined as follows:

(1) A lightweight and efficient convolutional sampling module, designated as Light C2f, is designed based on a multi-branch structure. This module can enhance the network’s capacity for multi-scale information perception, thereby facilitating a more accurate image representation through the multi-scale feature fusion.
(2) In order to ensure that the network has a global receptive field, a VMambaNeck architecture is put forward by incorporating the cross-scan module of VMamba into the original neck of YOLOv8-L.
(3) A dataset of strawberry flowers collected in natural environments on strawberry plantations is established.

## 2. Results

### 2.1. Ablation Experiments

Ablation experiments are widely used to compare the performances of the original and improved networks by gradually adding or removing certain components or functions. In order to verify the improved performance of VM-YOLO after each step, ablation experiments were also carried out on the strawberry flower dataset.

**Figure 1:**
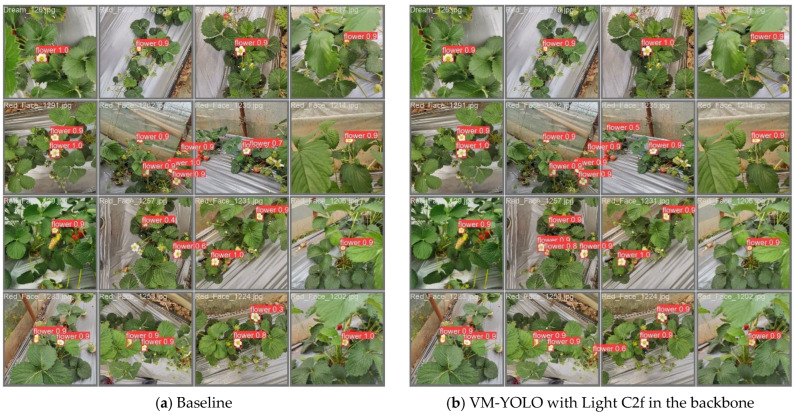

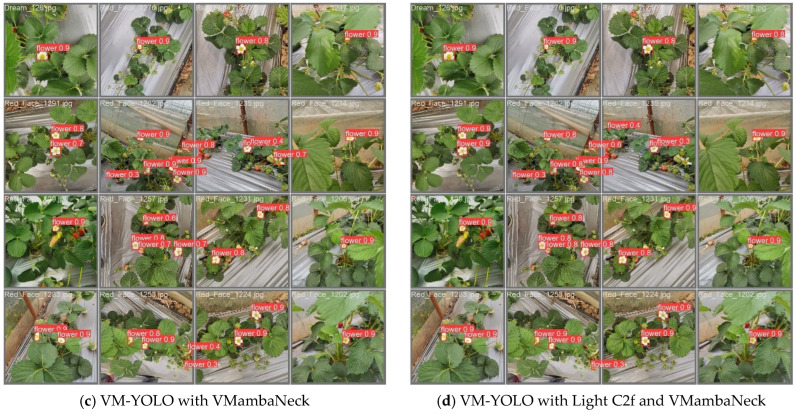
Predicted results of the ablation experiments. Note that the red dotted boxes represent correct features.

The numerical results of the ablation experiments are shown in Table 1, while the visualized predicted results are displayed in Figure 1. The VM-YOLO, with a modified backbone (using Light C2f modules to replace the C2f modules in the backbone of the baseline) and neck (introducing the VMamba network into the neck part of the baseline), achieved higher scores in all indicators, except for recall, compared to the baseline. Specifically, the mAP, speed, precision, and $F_{1}$ of VM-YOLO increased by 1.4%, 2.9 ms, 0.058, and 0.012, respectively, while params and GFLOPs decreased by 13 M and 67.6, with a slight decrease of 0.014 in the recall indicator. On the one hand, the performance can be enhanced, with only minor losses in recall and $F_{1}$, if the baseline is modified solely by using Light C2f modules to replace the C2f modules in the backbone. On the other hand, the scores of the indicators are also enhanced, with only minor losses in the precision and $F_{1}$, if the baseline is modified only with VMambaNeck. In general, the performance of VM-YOLO is significantly enhanced, particularly with regard to the substantial reductions in GFLOPs and params. This enhancement can be attributed to a reduction in redundant information. Consequently, the enhanced model is suitable for deployment on mobile platforms with limited computing capabilities in actual scenarios.

As illustrated in Figure 1, the original YOLOv8 model exhibited a higher recognition rate for strawberry flowers with frontal orientation posture. However, the probability of false and missed detections was augmented in scenarios involving relative clustering and slight occlusion of the strawberry flowers. When the algorithm was modified by introducing the VMamba network into the neck part of the baseline and focusing on non-positive strawberry flowers, the possibility of false and missed detections was also high. When the backbone and neck were, respectively, modified by Light C2f and VMambaNeck, the algorithm compensated for the limitations of the baseline, thereby enabling effective detection of strawberry flowers under relatively clustered and occluded conditions.

### 2.2. Comparison Results

#### 2.2.1. Comparison with Different Datasets

**Figure 2:**
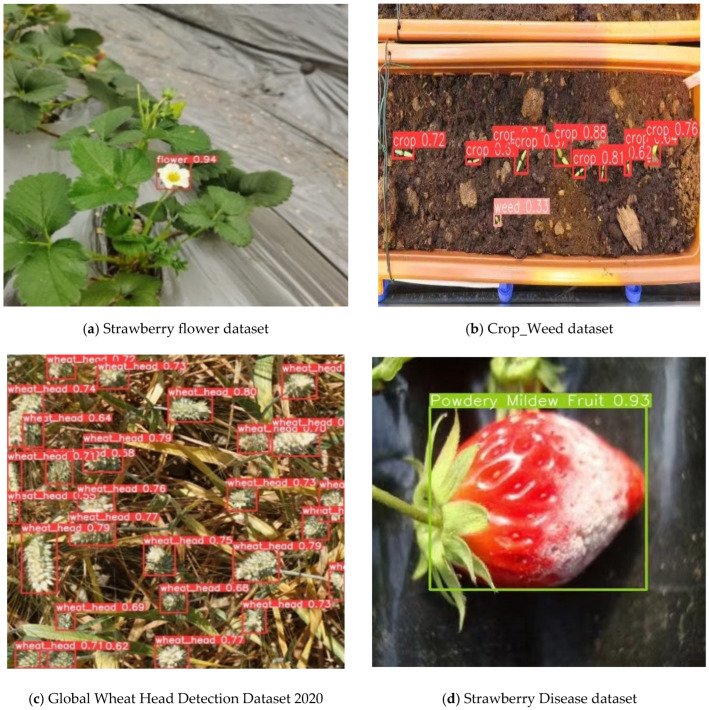
Visualization of the detection results of VM-YOLO on the different datasets. Note that the red and green dotted boxes both represent correct features.

The comparative studies were carried out on three different open-source datasets in the field of agriculture, including the Global Wheat Head Detection Dataset 2020, CropAndWeed dataset, and Strawberry Disease dataset, with the aim of further demonstrating the versatility of VM-YOLO. The results of the comparison are displayed in Table 2, and a visualization of the detection results is shown in Figure 2. It can be seen that VM-YOLO outperformed the baseline mainly in terms of the mAP, respectively, achieving a 3%, 2.8%, and 3.1% increase in the mAP for the Global Wheat Head Detection Dataset 2020, CropAndWeed, and Strawberry Disease datasets compared to the baseline. In addition, VM-YOLO outperformed the baseline in precision, recall, and $F_{1}$ score, except for a slight decline in precision observed on the CropAndWeed dataset. In summary, the results demonstrate that the VM-YOLO network exhibited superior performance in object detection tasks compared to the baseline.

#### 2.2.2. Comparison with Previous Works

With the rapid development of CNNs, numerous excellent object detection algorithms based on CNNs have been proposed by scholars. In order to evaluate the improved VM-YOLO in our work, its performance on the strawberry flower dataset was compared to that of four state-of-the-art object detection algorithms, as follows: YOLOv6 [32], Faster R-CNN [33], FCOS [34], and RetinaNet [35]. All of the experiments were executed on a uniform hardware platform, and each model was operated with an equal training/test set ratio. An expected training time of 500 was set for each model. Once trained, the weights that exhibited the best performance on the verification set were selected for testing using the test set. The mAP, speed of inference, params, and GFLOPs of the five object detection algorithms are shown in Table 3.

It can be seen that VM-YOLO performed the best in terms of the mAP, inference speed, and number of parameters among the five algorithms. VM-YOLO also had the second-best GFLOPs, only with slightly greater GFLOPs than that of Faster R-CNN. It is evident that the overall performance of VM-YOLO surpassed that of the other models, as evidenced by its high mAP, high inference speed, and low parameter number. Notably, the parameter number for VM-YOLO is merely 30 M, indicating that the improved model is both lightweight and suitable for implementation on platforms with limited computational capabilities in practical real-world scenarios.

## 3. Discussion

Many previous studies have been dedicated to enhancing the accuracy, lightweight, or detection speed of the object detection algorithms [36,37,38]. Nevertheless, considerable research has revealed an imbalance among the performance indicators. It is important to engage in trade-off optimization across several indicators, especially for agricultural machines which exhibit stringent requirements across all indicators while striving to maintain a low cost. With the consideration of these stringent requirements, we put forward a strawberry flower detection algorithm, named VM-YOLO, with the objective of achieving an equilibrium between accuracy and computational power. The VM-YOLO originated from YOLOv8-L has an optimized backbone network and a VMamba architecture-based neck. As a result, VM-YOLO achieved an mAP of 71.4% with only 30 M parameters, demonstrating excellent performance and promising deployment on low-power platforms.

Firstly, the performance improvements are mainly due to the use of Light C2f modules. As illustrated in Table 1, the modification of the backbone resulted in a substantial reduction in both parameters and GFLOPs. This phenomenon can be attributed to the CSPNet-based architecture of the Light Bottleneck, as illustrated in Figure 7. One of the lightweight improvement methods for algorithms involves the reduction in redundant gradient information, as it can lead to inefficient optimization and costly inference computations. The cross-stage feature fusion strategy and the truncating gradient flow in the CSPNet are well-suited to mitigate this problem [39]. Subsequently, the cross-stage feature fusion was executed on the input feature maps. One part of the feature maps underwent convolution operations, while the other part was directly spliced into the Light Bottleneck module. Furthermore, depthwise separable convolutions (DSCs) [40] were employed in the Light Bottleneck module. The DSC is composed of the following two constituent convolutions: DW_Conv and PW_Conv. The DW_Conv is responsible for spatial feature extraction, while the PW_Conv is responsible for channel feature extraction. Particularly, the PW_Conv utilizes a 1 × 1 convolution kernel to extract channel features, resulting in a significant reduction in parameter sharing among different channels. The DSC convolutional method facilitates the development of a lightweight algorithm.

Secondly, the improvements are partly attributed to the VMambaNeck, specifically, the VM-Block of VMambaNeck. The computational complexity of the network can be mitigated by the CSM module of VMamba. As shown in Figure 10, the CSM module initiates a four-way serialized scanning process of the image to ensure full correlation of the feature information among the feature maps. Subsequently, the scanning results are used to extract feature information via a state space model, which enables the allocation of weights dynamically based on the linear complexity calculation. Consequently, it improves the model’s ability to learn the contextual information of the image. The CSM module employs a four-way scanning technique to address the issue of orientation sensitivity, and enables the network to obtain the global receptive field through the calculation of linear complexity. The enhancement of the recall rate in Table 1 is partially attributable to the fact that the CSM module enables the model to have a global receptive field, which in turn mitigates the probability of missed detection for VM-YOLO.

**Figure 3:**
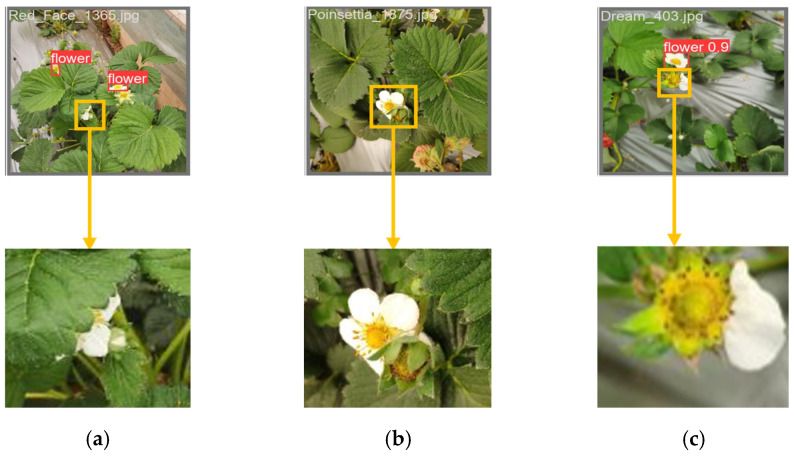
Fault detection results of the VM-YOLO on strawberry flowers. (**a**) Missed detection results at extreme angles. (**b**) Missed occlusion results under minor occlusion. (**c**) Missed test results due to petal defects.

Despite the advantages of VM-YOLO, there are still some potential problems when using it to detect strawberry flowers in the real environment. Due to the chaotic and random nature of strawberry flowers in actual growing conditions, it is difficult for agricultural machinery to take flower images from multi-angles. At the same time, some strawberry flowers may shed their petals, making detection even more difficult. For example, in cases of extreme severity, such as instances of severe occlusion, feature overlap, and partial feature loss, VM-YOLO is susceptible to missing detection targets. The three detection processes in which VM-YOLO missed strawberry flowers are shown in the yellow magnified area in Figure 3. Obviously, to effectively handle such extreme situations, further refinements to VM-YOLO are needed.

In subsequent studies, the generalization ability and robustness of neural networks can be enhanced from the following two perspectives: dataset augmentation and algorithm improvement. From the dataset perspective, artificial interference effects can be added to normal strawberry flower images, such as covering certain petals and simulating petal overlap, to enhance the model’s ability to recognize petal interference. Flower images with incomplete petals and images captured from extreme angles also can be added to the dataset to enhance the diversity and complexity of the dataset. From an algorithm improvement perspective, considering the possibility of feature integrity damage, the feature scanning path of the Cross-Scanning Module can be optimized to enhance the ability of the algorithm to learn incomplete features. This in turn increases the robustness of the model.

## 4. Materials and Methods

### 4.1. Image Dataset

**Figure 4:**
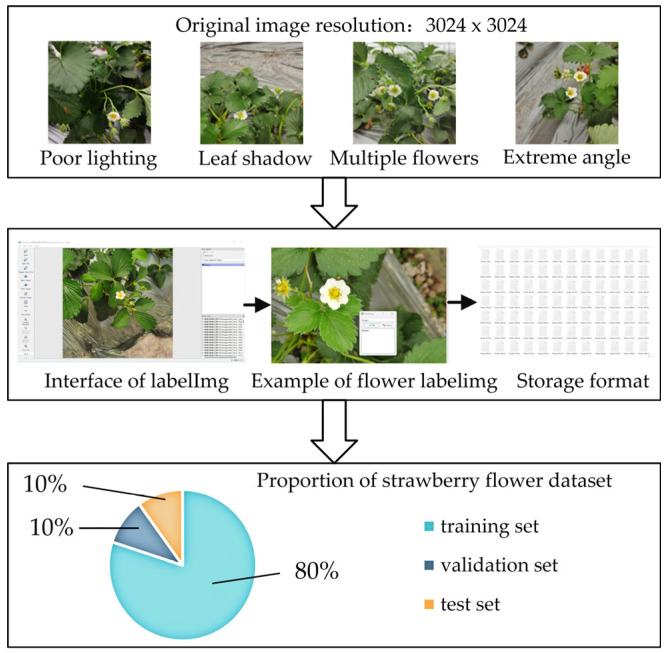
Establishment process of the strawberry flower dataset.

The process for establishing the strawberry flower dataset is illustrated in Figure 4.

Firstly, images of strawberry flowers, including Mengxiang, Red Face, and Santa Claus, were collected at Jiulongpo Strawberry Plantation in Chongqing, China (106°36′ E, 29°44′ N). The images were captured in a natural environment using a Xiaomi MI8 mobile phone with a resolution of 3024 × 3024 pixels in the *.jpg format. The collection time ranged from 2:00 P.M. to 5:30 P.M. in April 2023. The images were captured at distances ranging from 60 to 120 cm, which allows for sufficient design margins of height in the development of pollination robots. In other words, the images were collected under natural lighting conditions (full and low light, different angles, and different distances) and with natural growth characteristics (flower overlap, leaf occlusion, and incomplete flowers).

Secondly, the images were manually labeled with LabelImg software (v1.8.1) to ensure that the pistil of each flower was located at the center of the bounding box. The three varieties of strawberry patterns were marked as “flower”, and the label file was subsequently stored in the *.txt format. Each label file contained the image storage information, including the label rectangle box, image name, annotation name, and coordinate information. Eventually, the label files and images in the *.jpg format were saved in the YOLO format to construct a dataset consisting of 3388 images. Furthermore, during the training process, the flower images were processed using data augmentation strategies, including mosaic, mixup, random perspective, and HSV augmentation, in order to enhance the model’s generalization ability and robustness. It is important to note that the data augmentation does not modify the format of the images contained within the dataset, as the operations are executed online. Specifically, the images are loaded from the hard disk into the memory, where the color model of the images is converted from RGB to HSV for the purpose of HSV perturbation augmentation. Subsequently, the color model of the augmented images in the memory reverts to the RGB model.

Thirdly, the strawberry flower dataset was partitioned into three subsets at a ratio of 8:1:1: training set (2744 images), verification set (305 images), and test set (339 images). This ratio is considered reasonable for this small-scale dataset, as it has been demonstrated to be capable of balancing the training effect of the model and the evaluation accuracy [14,41]. A reasonable amount of data in the training set can provide the model with a sufficiently large number of samples for feature learning without causing overfitting. The number of flower images in each subset is presented in Table 4.

**Figure 5:**
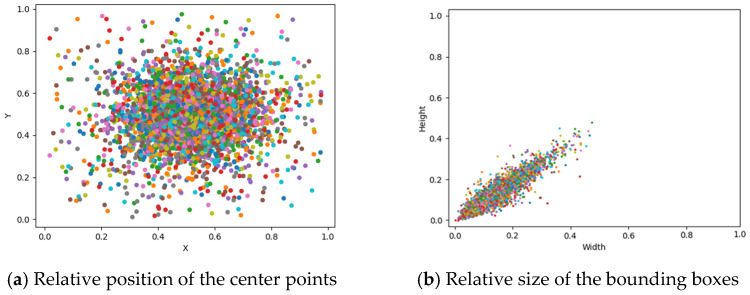
Scaling relationships between the bounding boxes and images. Note that the dots in different colors represent the samples, specifically the bounding boxes of the strawberry flowers in each image.

As shown in Figure 5, the scaling relationships between the bounding boxes and images were also analyzed following the establishment of the strawberry flower dataset. A bounding box, delineated by a rectangular frame, is generally employed to identify and locate objects of interest within an image by providing the object’s precise coordinate information. In order to ascertain the scaling relationships between the bounding boxes and images, the image sizes are scaled down to 1 × 1 in equal proportion. Then, the center points of the bounding boxes with respect to the images can be displayed, as shown in Figure 5a, while the sizes of the bounding boxes relative to the images can be displayed, as in Figure 5b. As illustrated in Figure 5a, the center points of the bounding boxes with respect to the images exhibit a clustered distribution within the range of 0.25 to 0.65 on both the X-axis and the Y-axis, with a limited number of points distributed outside of this range. This indicates that the center points of the strawberry flowers do not always correspond with the center of the images. As depicted in Figure 5b, the sizes of bounding boxes relative to the images are distributed diagonally, with the majority of bounding boxes falling within the range of 0.05 × 0.05 to 0.35 × 0.35. This phenomenon can be attributed to the fact that the detection objects are relatively small in comparison to the dimensions of the captured images. According to the scaling relationships between the bounding boxes and images, the objects exhibit a variety of center point locations and sizes. This can effectively enhance the generalization ability of the neural network.

In order to evaluate the versatility of the improvement network, three different open-source datasets in the field of agriculture, including the Global Wheat Head Detection Dataset 2020 [42], CropAndWeed dataset [43], and Strawberry Disease dataset [44], were selected for comparative studies. These three datasets offer researchers a range of perspectives for research and analysis due to the varying amounts of data contained therein. Specifically, the Global Wheat Head Detection Dataset 2020 is a large-scale dataset for wheat head detection, which consists of 4700 images of multiple varieties of wheat collected from different continents. The CropAndWeed dataset covers 74 relevant crop and weed species and provides over 8000 high-resolution images taken from real-world agricultural sites and specially cultivated outdoor plots of rare weed species. The Strawberry Disease dataset contains 2500 images of seven types of strawberry diseases. Each dataset was designed to fulfill distinct research requirements, and their specific data compositions and sample traits can provide technical backing for the exploration of a broader spectrum of technical application scenarios. By training the model on these datasets with different scales and characteristics, we can more accurately and comprehensively evaluate the generalizability of the model, as well as its adaptability and stability in different agricultural scenarios.

### 4.2. Improvement of YOLO

#### 4.2.1. Step 1: YOLO with Fast-Backbone

**Figure 6:**
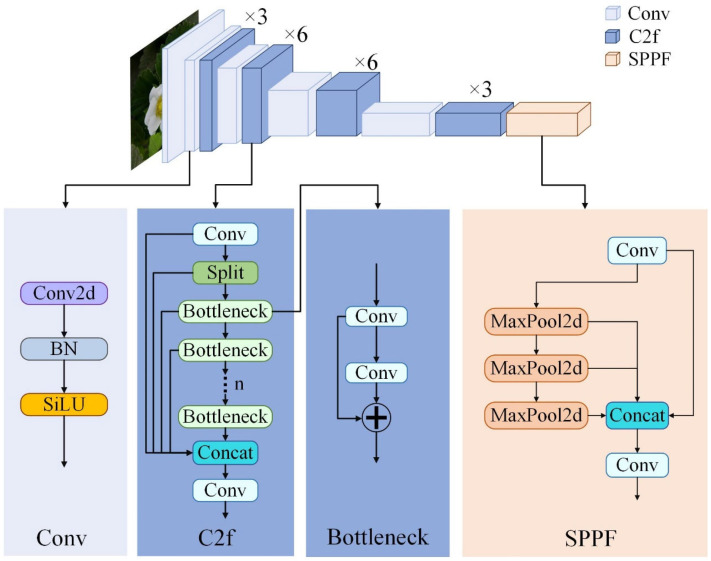
Backbone architecture of the original YOLOv8-L.

As shown in Figure 6, the backbone of the original YOLOv8-L (baseline) consists of the following three modules: Conv, C2f, and SPPF [45]. The horizontal flowchart in Figure 6 illustrates that the input image underwent a transformation from three channels (R, G, B) to multiple channels through successive layers of convolution. This architecture of the backbone is analogous to that of YOLOv5, with the notable distinction that YOLOv8 replaces YOLOv5’s C3 module with the Cross-Stage Partial Network (CSPNet)-based C2f module [46]. The C2f module has been demonstrated to significantly reduce the computational cost and number of parameters without compromising accuracy by connecting multi-bottleneck modules in a series to integrate gradient changes across the entire feature graph. In addition, the C2f module can be traced back to gradient-path-based ELAN (Efficient Long-Range Attention Network) [47] in YOLOv7. The employment of the C2f module enables YOLOv8 to reduce the model volume by regulating the number of stacked C2f modules without causing a rapid deterioration in model convergence due to changes in the model depth. Furthermore, the C2f module ensures that YOLOv8 obtains a more abundant gradient flow of information while maintaining its lightweight nature. At the end of the backbone, the SPPF module, comprising three serially connected maximum pooling layers with dimensions of 5 × 5, is employed to ensure the detection accuracy across diverse scales and the lightweight nature of the model.

**Figure 7:**
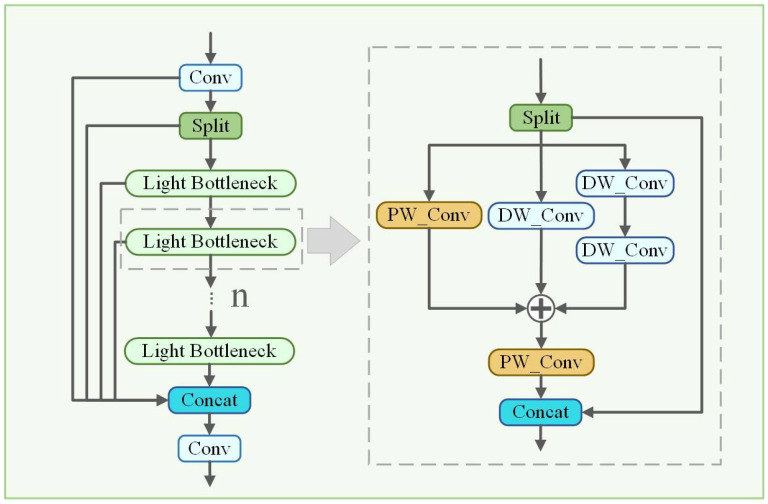
Framework of the Light C2f module.

In order to further reduce the model volume and computational requirements, a Light C2f module is proposed in this paper. As shown in Figure 7, the Light C2f module retains the same framework as the original C2f, yet differs in that it replaces the Bottleneck of C2f with a CSPNet-based Light Bottleneck. The features extracted from the convolutional layer can be reused by the stacked Light Bottleneck, thereby reducing the redundant information, number of parameters, and computational power demand of the backbone network. Inspired by the Inception network paradigm, the Light Bottleneck is designed as a multi-branch convolution architecture to further enhance the ability to perceive objects at different scales. Specifically, the pointwise convolution (PW_Conv) and depthwise convolution (DW_Conv) are arranged in parallel within the Light Bottleneck. In comparison to conventional convolution, PW_Conv and DW_Conv possess fewer parameters and lower operational costs. Consequently, better image representation can be obtained by fusing features with varying scales of images extracted by multiple convolution kernels.

#### 4.2.2. Step 2: VMamba in the Neck of YOLO

**Figure 8:**
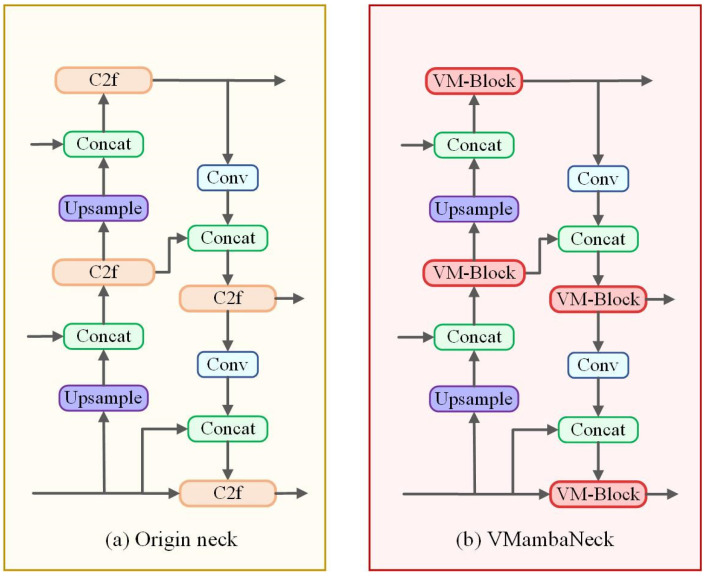
Original neck and VMambaNeck.

As shown in Figure 8a, the original neck architecture is constructed based on the integration of an FPN [48] and PAN [49], thereby facilitating the cross-scale fusion of feature maps at different scales for effective information exchange. Specifically, an FPN conveys the semantic features of deep feature maps to the shallow layers, enhancing the semantic expression of the shallow feature maps. Conversely, PAN transmits the location information from the shallow feature maps to the deep layers, thereby improving the location capabilities of the deeper layers. Furthermore, a considerable number of C2f modules are employed in the original neck to improve the accuracy and generalization ability of the algorithm, because they can take full advantage of both location and semantic information at different scales. However, the C2f model relies on a substantial number of traditional fixed receptive field-based convolution methods, which exhibit a lack of attention to global information, thereby diminishing the network’s capacity to capture features at different scales.

To address the aforementioned issues, we propose an improved neck network named VMambaNeck, as shown in Figure 8b. The primary distinction between the original neck and the VMambaNeck lies in the substitution of the C2f module in the former with the VM-Block, which is derived from the VMamba network.

The fundamental concepts of the VM-Block are derived from the state space model (SSM), which is favorable for describing and analyzing the behavior of a dynamic system. It maps system inputs, $x(t)\in R^{L}$, to responses, $y(t)\in R^{L}$, and can be described by the following differential equation:

(1) $$\left\{\begin{array}{c} h\prime (t)=Ah\left(t\right)+Bx(t)\\ y\left(t\right)=Ch\left(t\right)+Dx(t) \end{array}\right.$$

where h(t) represents the state vector of the system; x(t) and y(t) represent the input and output vectors, respectively; $A\in C^{N\times N}$ represents the state transition matrix; $B\in C^{N}$ and $C\in C^{N}$ represent the input and output matrices, respectively; $D\in C^{1}$ represents the direct transfer matrix; and N represents the number of variables in the state space.

State space models are conventionally employed to deal with sequential data in the deep learning field, because the long-term dependencies of the data can be more effectively captured by mapping sequential data to state spaces. In view of the discrete sequential data, the continuous function shown in Equation (1) must be discretized. With the assumption that the input vector $x_{k}\in R^{L\times D}$ is a D-dimensional signal stream with a length of *L*, Equation (1) can be discretized as follows:

(2) $$\begin{array}{c} h_{k}=\bar{A}h_{k-1}+\bar{B}x_{k}\\ y_{k}=\bar{C}h_{k}+\bar{D}x_{k}\\ \bar{A}=e^{∆A}\\ \bar{B}=\left(e^{∆A}-I\right)A^{\left(-1\right)}B\\ \bar{C}=C \end{array}$$

where B  $\in R^{D\times N}$, $C\in R^{D\times N}$, and $∆\in R^{D}$.

In fact, first-order Taylor series expansion is usually used for linear approximation of $\bar{B}$ to simplify the algorithm. Then, $\bar{B}$ can be expressed as follows:

(3) $$\bar{B}=(e^{\Delta A}-I)A^{-1}B\approx (\Delta A)(\Delta A{)}^{-1}\Delta B=\Delta B$$

**Figure 9:**
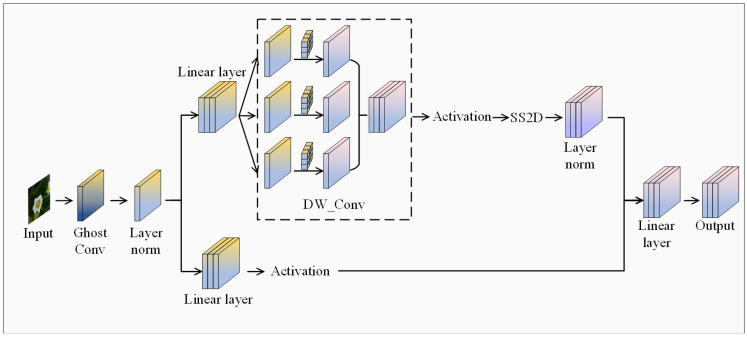
Framework of the VM-Block.

As illustrated in Figure 9, the SSM-based VM-Block is divided into two information flows subsequent to Chost convolution and the linear embedding layer. One branch enters the core module SS2D via the DW_Conv module and the Silu activation function. Subsequent to the process of standardization, it combines with the other information flow and eventually outputs through the linear layer. The introduction of the VM-Block effectively addresses the issue of the fixed receptive field of the traditional convolution method in the C2f module.

**Figure 10:**
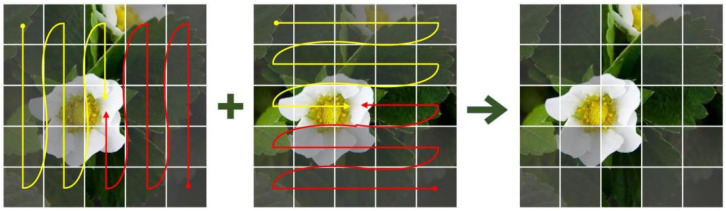
CSM-based scanning mode.

In the VM-Block, the 2D feature maps acquired by the DW_Conv module must be flattened and scanned in sequence in the subsequent step. However, if the 2D feature maps are flattened and scanned directly, the global receptive field will be lost because of the non-causal property of them. To address this challenge, a 2D selective scanning method, designated as the Cross-Scan Module (CSM), is employed within the VMamba network. As illustrated in Figure 10, CSM-based scanning is performed in different directions from the four corner pixels of the image.

**Figure 11:**
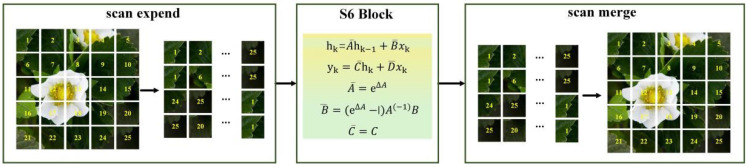
Flow diagram of the SS2D.

In the SS2D module, the cross-scanning results based on the CSM were initially serialized and, subsequently, selectively scanned using the state space model. Finally, the outputs of the state space model are recovered and integrated to generate an image. The flow diagram of SS2D is depicted in Figure 11.

**Figure 12:**
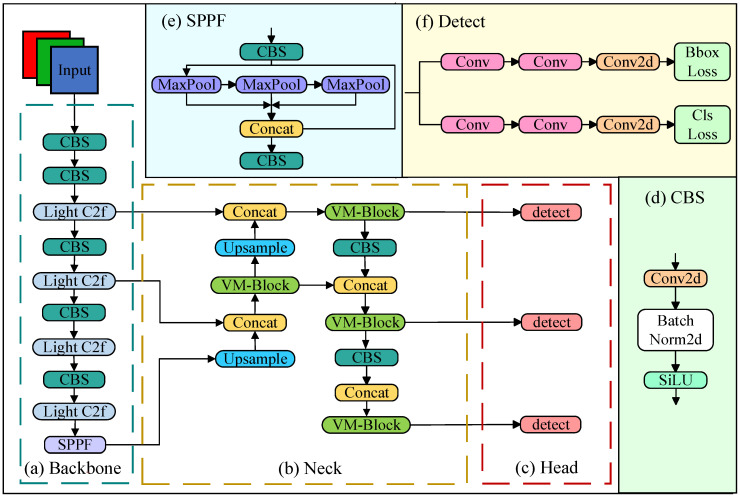
Framework of the VM-YOLO.

Following the improvements in the backbone and neck, the framework of VM-YOLO is illustrated in Figure 12. The functions of the main modules are described as follows:

(1) The CBS (2D convolution, batch normalization, and SiLU) module, which is the most prevalent structure observed in the YOLOv8 model, consists of a 2D convolution layer, batch normalization layer, and SiLU activation function. The term “2D” indicates that the convolution is applied to the following two spatial dimensions: height and width.
(2) The SPPF (spatial pyramid pooling-fast) module is a computational framework that can execute pooling operations on input images across diverse scales. This process facilitates the acquisition and integration of multi-scale feature information, such as the shape, size, and position information regarding the object, thereby enhancing the accuracy of the object detection.
(3) The Detect module is composed of two parallel tracks. One track is dedicated to boundary box prediction, while the other is employed for class prediction. Each of these tracks contains two separate convolution layers and a 2D convolution layer. Ultimately, the boundary frame loss and class loss are calculated, respectively.

Compared to YOLOv8, our work has the following distinctions:

(1) Firstly, the multi-branch architecture-based Light C2f module is utilized to replace the C2f module in the backbone part of the baseline. This replacement reduces the redundant information, number of parameters, and computational power demand while improving the ability to perceive objects at different scales.
(2) Secondly, the state space model-based VM-Block of the VMamba network is introduced into the neck part of the baseline with the objective of solving the fixed receptive field problem in the C2f module of traditional convolution methods.

The main process of the VM-YOLO algorithm is shown in Algorithm 1.


**Algorithm 1.** Main process of VM-YOLO.**Input**: images and labels from the strawberry flower dataset **Output**: target position
# *Preprocess: customize mosiac, HSV, and other image enhancement methods in the dataset*
dataset = Dataset(image, label)
data = DataLoader(dataset, batch, shuffle = True)
# *Construct model*
Model = VM-YOLO()
# *Define the loss function and optimizer*
Loss = YOLOV8Loss()
Optimizer = SGD()
**for** epoch in range(epochs):
  Model.train()
  **for** i, (images, labels) in enumerate(data):
    # *Copy data from memory to GPU*
    images = images.to(device)
    labels = labels.to(device)
    # *The model performs forward propagation*
    outputs = Model(images)
    # *calculate the loss*
    loss = YOLOv8Loss(outputs, labels)
    # *Propagate back and calculate the gradient*
    optimizer.zero_grad()
    loss.backward()
    optimizer.step()
  **End for**
**End for**
Model.eval()
# *Model inference*
with torch.no_grad():
  image = image.to(device)
  prediction = Model(image)
# *The results are postprocessed to obtain the exact target position*
postprocessing(prediction)
**Return** target position


### 4.3. Model Training and Evaluation

In order to verify the performance of VM-YOLO in detecting strawberry flowers, a comparison was made with the baseline and other object detection networks. The parameters of the simulation platform utilized for the training and testing are shown in Table 5.

Subsequently, the network was trained using the images in the dataset. In order to ensure the universality and reproducibility of our work, a unified processing method was adopted for the subsequent datasets. The training parameters are shown in Table 6.

The indicators used to evaluate the performance included precision (*P*), recall (*R*), $F_{1}$, mAP, speed of inference, params, and GFLOPs.

Among the indicators, *P* indicates the proportion of true positive samples in all predicted positive samples, *R* can be used to reflect the proportion of predicted true positive samples in all actual positive samples, $F_{1}$ is a comprehensive index used to measure the precision and recall, and mAP represents the mean average precision of the object detection with an IOU threshold of 0.5. The formulas for calculating *P*, *R*, $F_{1}$, and mAP are shown in Equation (4), as follows:

(4) $$\begin{array}{c} P=\frac{TP}{TP+FP}\\ R=\frac{TP}{TP+FN}\\ F_{1}=2\frac{P\times R}{P+R}\\ mAP=\frac{\sum_{C=1}^{C}AP\left(c\right)}{C} \end{array}$$

where T P, FP, and FN represent the numbers of true positive, false positive, and false negative cases, respectively; C represents the number of detection categories, with C=1 in this work.

## 5. Conclusions

Accurately and rapidly detecting flowers at a low computational cost is an important step in intelligent orchard management, including tasks such as yield estimation, robotic pollination, and flower thinning. Therefore, this paper proposed an improved object detection algorithm (called VM-YOLO) based on YOLOv8-L with VMamba for strawberry flower detection, with the aim of deploying it in automatic pollination devices in the future. After the training and testing of VM-YOLO on the strawberry flower dataset, we compared it with the baseline and other algorithms on both the strawberry flower dataset and other open-source datasets. The conclusions are as follows:

(1) The improved VM-YOLO network was developed by modifying the backbone and neck of YOLOv8-L. First, the Light C2f module, which has a multi-branch convolution architecture, was used to replace the C2f module in the backbone of the baseline to improve the network’s perception of multi-scale information. Second, the neck of baseline was modified by introducing the state space model-based VMamba network, which can be used to effectively solve the fixed receptive field problem of traditional convolution methods in the C2f module.
(2) The experimental results show that the VM-YOLO algorithm can be effectively used for object detection. Compared to YOLOv6, Faster R-CNN, FCOS, and RetinaNet, the VM-YOLO algorithm achieved the highest performance in terms of the mAP, inference speed, and number of parameters, with only a slight increase in GFLOPs compared to the Faster R-CNN. This indicates that the VM-YOLO has a smaller model size, fewer network parameters, lower computational usage, and faster detection speed while maintaining high detection performance.

In the future, the strawberry flower dataset is planned to be expanded by increasing the number of strawberry flower varieties and adding strawberry flowers with incomplete petals or over-obscuration. The expanded dataset can lead to a better training effect, as well as a better generalization ability of the algorithm. In addition, the oblique and inverse oblique scan paths can be integrated into the Cross-Scanning Module. The increase in scan paths is beneficial to improve the algorithm’s feature extraction, especially when the object has incomplete features. Although the VM-YOLO is proposed for strawberry flower detection, its universality allows for it to be extended to other objects in the agricultural field. In the future, by constructing multi-task datasets, it is expected to create an automated agricultural production management system that can integrate the functions of pest detection, weed identification, and fruit flower detection, thus facilitating agricultural production.

## Figures and Tables

**Table 1:** Numerical results of ablation experiments.

Model	Modules		Results						
	Light C2f	VMambaNeck	mAP	Speed	Params	GFLOPs	Precision	Recall	$F_{1}$ Score
Baseline			70.0%	25.3 ms	43 M	165.7	0.813	0.637	0.714
VM-YOLO	√		71.2%	19.6 ms	31 M	106.4	0.862	0.600	0.708
VM-YOLO		√	70.5%	23.5 ms	42 M	157.0	0.756	0.662	0.706
VM-YOLO	√	√	71.4%	22.4 ms	30 M	98.1	0.871	0.623	0.726

**Table 2:** Comparison results with the different datasets.

Datasets	Model	mAP	Precision	Recall	$F_{1}$ Score
Global Wheat Head Detection Dataset 2020	Baseline	63.2%	0.751	0.584	0.657
	VM-YOLO	66.2%	0.793	0.596	0.681
CropAndWeed	Baseline	72.9%	0.711	0.758	0.734
	VM-YOLO	75.7%	0.708	0.766	0.736
Strawberry Disease	Baseline	74.9%	0.800	0.697	0.745
	VM-YOLO	78.0%	0.830	0.726	0.775

**Table 3:** Comparison results for the strawberry flower dataset.

Model	mAP	Speed	Params	GFLOPs
Yolov6	65.5%	23.3 ms	110 M	391.20
Faster R-CNN	59.3%	29.9 ms	41 M	91.30
FCOS	60.0%	87.3 ms	90 M	175.00
RetinaNet	62.7%	30.0 ms	38 M	95.53
VM-YOLO	71.4%	22.4 ms	30 M	98.10

**Table 4:** Strawberry flower datasets.

Dataset	Variety	Number	Total
Training set	Mengxiang	635	2744
	Red Face	1214	
	Santa Claus	895	
Validation set	Mengxiang	72	305
	Red Face	132	
	Santa Claus	101	
Test set	Mengxiang	93	339
	Red Face	142	
	Santa Claus	104	

**Table 5:** Parameters of the simulation platform.

Hardware	Parameter
Main board	ASUS WSX299
CPU	Intel (R) Core (TM) i9-10940X
RAM	64 G
GPU	GEFORCE RTX3090 Ti 24 GB
Operating system	Ubuntu 18.04

**Table 6:** Training model parameter settings.

Parameter	Value
Input image size	640 × 640
Learning rate scheduler	Linear LR
Factor	0.01
Initial learning rate	0.01
Optimizer	SGD
Momentum	0.987
Patience	50
Batch_size	16
Epoch	500

## Data Availability

The datasets can be found at the following: The strawberry flower dataset (Accessed on 2 February 2024): https://drive.google.com/drive/folders/1aT6ur3cLPp0xD0urIH6ex_mrFYkIAtm8; The Global Wheat Head Detection Dataset 2020 (Accessed on 10 February 2024): http://www.global-wheat.com/gwhd.html; The CropAndWeed dataset (Accessed on 15 February 2024): https://github.com/cropandweed/cropandweed-dataset; and The Strawberry Disease dataset (Accessed on 20 February 2024): www.kaggle.com/usmanafzaal/strawberry-disease-detection-dataset.
